# Work outcomes of breast cancer survivors who returned to work after treatment: CANTO cohort

**DOI:** 10.1093/eurpub/ckac131.211

**Published:** 2022-10-25

**Authors:** G Ruiz-de-Azua, I Kousignian, I Vaz-Luis, E Caumette, A Di Meglio, J Havas, E Martin, AL Martin, A Dumas, G Menvielle

**Affiliations:** 1 Institute Pierre Louis Epidemiology and Public Health, Sorbonne University, INSERM, Paris, France; 2 BioSTM - UR 7537, Université Paris Cité, Paris, France; 3 Medical Oncology Department, Gustave Roussy, Villejuif, France; 4 INSERM Unit 981, Gustave Roussy, Villejuif, France; 5 UNICANCER, Paris, France; 6 ECEVE, UMR 1123, Université de Paris, Paris, France

## Abstract

**Background:**

As survival rates among breast cancer patients improve there is an increasing need to address breast cancer survivors’ (BCS) issues, professional life being a key aspect. Return to work (RTW) of BCS has been largely studied, but studies on job maintenance and its determinants are scarce. We aim to study job maintenance after RTW and the associated factors among BCS.

**Methods:**

We used data from the CANTO cohort, a French prospective cohort of BCS. We included 1643 BCS aged <57 at diagnosis (dx) who returned to work two years after dx. We excluded self-employed BCS. Using multinomial logistic models, we assessed the association between activity status one year after they return to work. (i.e. active, sick leave, or unemployed, retired or invalidity) and sociodemographic, clinical, health status and work-related factors.

**Results:**

Overall, 87% of BCS were active, 10% were on sick leave and 3% were on unemployment, retirement or invalidity one year after they return to work. In the fully adjusted model being on sick leave was associated with stage III at dx (OR: 1.89, 95% CI: 1.11-3.22), being severely fatigued at the moment of returning to work (OR: 1.53, 1.04-2.27), and having workplace accommodations (OR: 1.79, 1.14-2.81). The unemployed, retired, invalidity status was negatively associated with professional life being more than or as important as one’s personal life (OR: 0.51, 0.26-0.98) and being <50 years old (OR: 0.51, 0.27-0.96), and positively associated with having a fixed-term contract (OR: 2.69, 1.39-5.18) and working for a small company (OR: 2.73, 1.24-6.02).

**Conclusions:**

A non-negligible proportion of BCS are non-active one year after they return to work. While clinical factors are associated with sick leave, work related factors are associated with the unemployed, retired, and invalidity status. RTW should not be regarded as the ultimate goal and future policies should focus on ensuring people are ready to return to work and maintain their jobs.

**Key messages:**

• A non-negligible proportion of breast cancer survivors are non-active one year after they return to work.

• Future policies should ensure job maintenance along with return to work.

